# Comment on Eschweiler et al. Anatomy, Biomechanics, and Loads of the Wrist Joint. *Life* 2022, *12*, 188

**DOI:** 10.3390/life12081166

**Published:** 2022-07-31

**Authors:** Pantelis T. Nikolaidis, Jose Afonso

**Affiliations:** 1 School of Health and Caring Sciences, University of West Attica, 12243 Athens, Greece; 2 Centre for Research, Education, Innovation and Intervention in Sport, Faculty of Sport, University of Porto, 4200-450 Porto, Portugal; jafonsovolei@hotmail.com

We have read ‘Anatomy, biomechanics and loads of the wrist joint’ [1] with great interest, and have found that it is an important contribution on the field of anatomy, biomechanics and kinesiology. In their 17-page article, Eschweiler et al. [1] successfully and concisely present information about the bones of the wrist joint and the way they articulate, including the role of ligaments, the skeletal muscles of this joint and their functions. Thus, the information provided in their paper would appeal to researchers and scientists in the field of anatomy, biomechanics and kinesiology. An important point addressed by their paper is the interindividual variability in the relevant information. There is extensive person-to-person variability in terms of wrist and finger muscles and ligaments, which poses clinical and functional implications. A few examples included the flexor carpi radialis (which may be absent), radial head of the flexor digitorum superficialis (which may be absent or may not reach the little finger), palmaris longus (which is often absent on one or both sides), flexor pollicis longus (which may be connected to the flexor digitorum superficialis or profundus, or the entire muscle may be absent), extensor carpi radialis longus (which may mesh with intermetacarpal ligaments), extensor carpi radialis longus and brevis (which may be united or share muscular slips), tendons of the extensor digitorum (which may be variably deficient, doubled, or tripled, and occasionally may also reach the thumb), extensor digiti minimi (which may be fused with the extensor digitorum), extensor pollicis brevis (which may be absent or fused with the abductor pollicis longus), and extensor indicis (which occasionally also reaches additional fingers) [2]. The aim of the present letter-to-the-editor is to highlight a few aspects that were not fully discussed in the paper of Eschweiler et al. [1], and their consideration may compliment this work: (a) the extrinsic muscles of the fingers, and (b) the position of the elbow and finger joints.

The second figure in Eschweiler et al. [1] provides an overview of the six muscles acting only in the wrist and not in the finger joints (flexor carpi radialis, palmaris longus, flexor carpi ulnaris, extensor carpi radialis longus, extensor carpi radialis brevis and extensor carpi ulnaris: the ‘wrist muscles’) with regard to their size as well as their moment arms. It is a very informative figure, through which one can understand which is the ‘stronger’ muscle as well as which muscle crosses away from the axis of motion, and consequently, has the advantage of an optimal moment arm. However, the moment arm alone is not enough to assess the contribution to movement, as the cross-sectional area and motor coordination also play important roles [3]. In addition to the six ‘wrist muscles’, the three finger muscles (flexor digitorum superficialis, extensor digitorum and abductor pollicis longus) and their actions are also reported in the accompanying text, suggesting that finger muscles act on the wrist joint, too. At this point, we consider that all extrinsic finger muscles and their actions on the wrist joint should be discussed in more detail. Thus, the role of the flexor pollicis longus, extensor pollicis longus, extensor pollicis brevis, flexor digitorum profundus, extensor digiti minimi and extensor indicis should also be presented and discussed [2]. Concerning wrist motion, we would add circumduction to the reported flexion, extension, adduction and abduction; because the wrist joint has two degrees of freedom, it may perform a combination of the reported motions. The maximum range of motion and force production depend on elbow flexion/extension and supination/pronation.

With regards to the action of the ‘wrist muscles’ and extrinsic finger muscles, it should be highlighted that all of them are multi-articular because they cross not only over the wrist but also over the elbow and/or finger joints [2]. Thus, their action on the wrist joint, as well as the range of motion of this joint, depends on the position of the nearby joints. This will change their moment arms, and also their degree of shortening or lengthening, which will modify their ability to produce force [3]. Because the paper by Eschweiler et al. [1] discusses the loads on the wrist joint, it is noteworthy that these loads and the range of motion vary as a function of the nearby joints. For instance, flexing all of the finger joints results in (a) the passive inadequacy of finger extensors (they stretch in finger joints, limiting their capacity to lengthen further in the wrist joint and consequently restricting the physiological range of motion of wrist flexion), and (b) the active inadequacy of finger flexors (they contract in finger joints, limiting their capacity to contract maximally in the wrist joint and consequently decreasing the force output capacity in the wrist joint. Another example is the extensor carpi radialis longus, which plays a minimal role in elbow flexion in addition to its role in the wrist joint (wrist extension and radial deviation). Thus, flexing the elbow is expected to weaken wrist extension due to the active inadequacy of the extensor carpi radialis longus. Moreover, extending the wrist joint improves the ability to forcefully flex the finger joints, as it avoids the excessive shortening (and consequent active inadequacy in force production) of the finger flexors [3]. This is important, for example, when performing power grips during resistance training exercises with high loads (e.g., pull-ups, lat pull-downs). We also agree with the statement of the authors that knowing wrist anatomy is decisive in the proper treatment of wrist injuries. To enhance this statement, because many wrist injuries derive from neural or vascular entrapment [2], and these may originate more proximally (e.g., at the elbow level or in the interosseous membrane of the forearm), focusing excessively on the wrist may neglect non-local origins of problems, which would compromise both diagnosis and treatment.

The present letter-to-the-editor is intended to highlight some aspects that describe the interaction of the wrist with the nearby joints. It is acknowledged that for educational purposes (e.g., teaching kinesiology to undergraduate students), providing information on each joint separately is a widely used methodological approach followed by several kinesiology textbooks [3,4,5,6]. A localized vision may impair the understanding of the whole and, when multi-articular muscles are present, this brings about severe limitations in the understanding of functionality. In such a context, it is a real challenge to explain the role of multi-articular muscles, such as finger muscles, not only with regard to their actions on the joint where the focus is, but also on all other joints on which they act as well. From this point of view, we recommend the paper of Eschweiler et al. [1] for further reading by a broad readership, and encourage the authors to develop the interactions among joints in their future works.
